# Health-related quality of life-based definition of remission from depression among primary care patients

**DOI:** 10.3389/fpsyt.2023.926410

**Published:** 2023-03-27

**Authors:** Kirsi Riihimäki, Harri Sintonen, Maria Vuorilehto, Erkki Isometsä

**Affiliations:** ^1^Mental Health Research Unit, National Institute for Health and Welfare, Helsinki, Finland; ^2^Department of Psychiatry, University of Helsinki and Helsinki University Hospital, Helsinki, Finland; ^3^Department of Public Health, University of Helsinki, Helsinki, Finland

**Keywords:** depression, remission, health-related quality of life, 15D, HAMD, BDI

## Abstract

**Background:**

Depression undermines health-related quality of life (HRQoL). Remission is the central aim of all treatments for depression, but the degree of remission necessary for depressive patients’ HRQoL to correspond to the normal range of the general population remains unknown.

**Methods:**

The Vantaa Primary Care Depression Study prospectively followed-up a screening-based cohort of depressive primary care patients for 5 years. The Structured Clinical Interview for DSM-IV Axis I Disorders (SCID-I) was used to diagnose major depressive disorder. HRQoL was measured by the generic 15D instrument at baseline and at 5 years (*N* = 106, 77% of baseline patients), and compared with the 15D results of an age-standardized general population sample from the Finnish Health 2011 Survey (*N* = 4,157). Receiver operating characteristic analyses determined the optimal Hamilton Depression Rating Scale (HAMD), Beck Depression Inventory (BDI), and Beck Anxiety Inventory (BAI) cut-offs for remission, using the 15D score as the construct validator. Remission was defined as the score at which HRQoL reached the general population range (minimum mean − 1 SD). As age may influence HRQoL, patients older and younger than the median 52 years were investigated separately.

**Results:**

For HAMD, the optimal cut-off point score was 8.5, for BDI 10.5, and for BAI 11.5. The differences between the findings of the younger and older patients were small.

**Limitations:**

Cross-sectional analysis, small number of patients in the cohort.

**Conclusion:**

Depressive primary care patients’ HRQoL reaches the normal variation range of the general population when their depression and anxiety scores reach the conventional clinical cut-offs for remission.

## 1. Introduction

National treatment guidelines commonly recommend that the goal of treatment for depression is remission. However, remission can be defined from multiple valid perspectives. For assessing the severity of depressive symptoms, the Hamilton Rating Scale for Depression (HAMD) (1) is a widely used observer-rated scale, with a HAMD score of ≤7 being the conventional operational definition for remission in clinical studies. This may not be sufficiently stringent if remission is defined as depressive symptoms so mild that they pose no significant risk of recurrence (2). The discussion on optimal symptomatic remission measures, their cut-off points, and their heterogeneity is ongoing (2–4).

One important perspective of this debate concerns health-related quality of life (HRQoL), which comprises the physical, mental and social components affected by illnesses and treatments. Depressive disorders impair HRQoL to an even greater extent than the most common chronic physical diseases (5–7). An important question is how completely depressive symptoms must be alleviated for HRQoL to correspond to the normal range of the general population. However, only limited data are available (2, 8), and we unaware of any primary care studies that have examined remission in terms of HRQoL.

In our earlier study, we found that HRQoL differed from that of the general population even among depressive patients who apparently attained full clinical remission (9). Here, our aim was to examine the optimal cut-off points of depression scales in terms of HRQoL. As comorbidity with anxiety disorders predicts the outcome of depression and influences HRQoL (9), we also evaluated an anxiety scale.

## 2. Method

### 2.1. Vantaa primary care depression study

**Figure 1:**
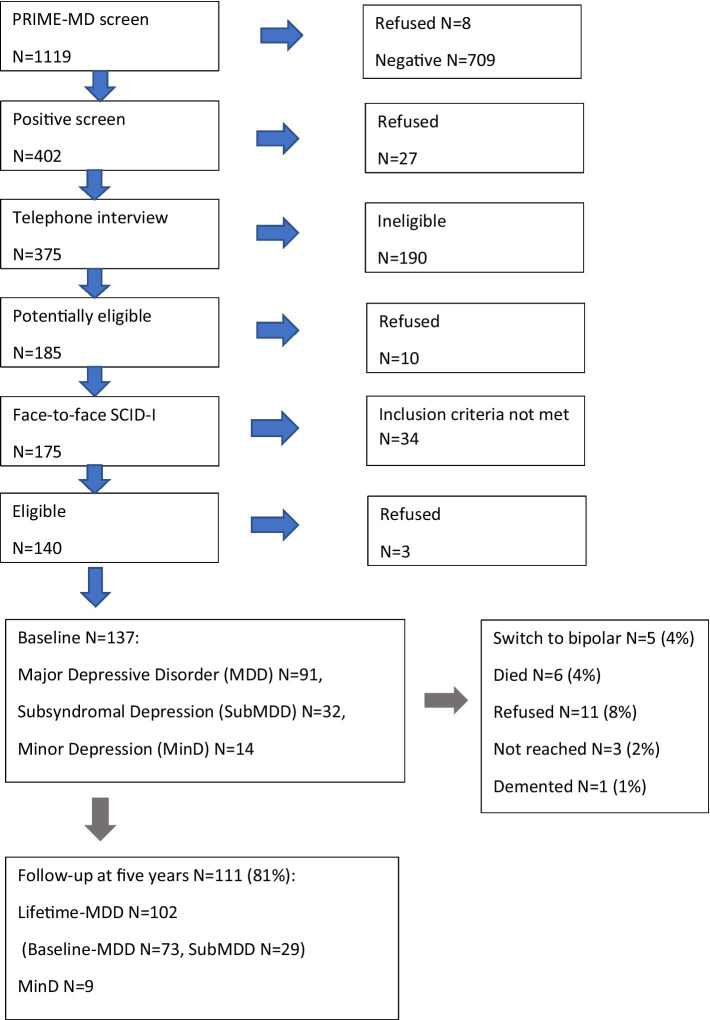
Flow chart of the screening, diagnostic interviews and follow-up of the Vantaa Primary Care Study (PC-VDS).

The Vantaa Primary Care Depression Study (PC-VDS) is a collaborative research project between the National Institute of Health and Welfare, the University of Helsinki and the City of Vantaa, Finland. Details of the methodology and patient characteristics have been published elsewhere (10, 11). Flow chart of patient sampling and follow-up of the study is presented in Figure 1.

In brief, based on stratified sampling in 2002, 402 of 1,111 consenting general practitioners’ patients aged 20–69 in the city of Vantaa, Finland, had a positive Primary Care Evaluation of Mental Disorders (PRIME-MD) screen for depression. Altogether 375 patients were interviewed by telephone, where the presence of at least one core symptom of major depressive disorder (MDD) according to the Structured Clinical Interview for DSM-IV Axis I Disorders (SCID I/P) [21] was confirmed. All 175 potentially eligible consenting patients were interviewed face-to-face using the SCID I/P. The diagnostic reliability for current depressive disorder diagnoses was excellent (kappa = 1.0). Inclusion criteria were current (1) major depressive disorder (MDD), (2) dysthymia, (3) subsyndromal MDD with two to four depression symptoms (minimum one core symptom) and lifetime MDD or (4) minor depression similar to subsyndromal MDD but without MDD history. Patients who refused to participate (15%) did not differ significantly in age or gender from those who consented (10).

The final PC-VDS study sample comprised 137 patients. Current and lifetime psychiatric disorders were assessed using SCID-I/P and SCID-II interviews for DSM-IV, including a careful evaluation of psychiatric and somatopsychiatric comorbidity (10). The severity of depressive symptoms was measured using the observer-rated HAMD (1) and the self-reported Beck Depression Inventory (BDI) (12). The severity of anxiety symptoms was measured using the self-reported Beck Anxiety Inventory (BAI) (13).

During follow-up, using a graphic life chart, time after the baseline interview was divided into three categories in accordance with the DSM-IV: state of (1) major depressive episode (MDE) (five or more of the nine criteria symptoms); (2) partial remission (one to four symptoms); or (3) full remission (no symptoms). Of the patients, 112 completed the five-year follow-up assessments, which included the same diagnostic interviews, scales, and medical and psychiatric records as the baseline investigation (11). Drop-outs (18%) did not differ from participants in age, gender, baseline depression severity (11) or 15D score.

At baseline (2002–2003) and at 5 years (2007–2008), HRQoL was measured using a generic, self-administered and preference-based tool the 15D, which can be used as a profile and a single index score measure. The health state descriptive system (questionnaire) is composed of the following 15 dimensions: mobility, vision, hearing, breathing, sleeping, eating, speech (communication), excretion, usual activities, mental function, discomfort and symptoms, depression, distress, vitality, and sexual activity, each having five ordinal levels. The single index score (15D score), representing the overall HRQoL on a scale of 0–1 (1 = full health, 0 = dead) is calculated from the questionnaire using a set of population-based preference or utility weights (14). In the important properties (reliability, validity, discriminatory power, responsiveness to change), the 15D is at least equally effective as the other preference-based generic instruments (15, 16). The 15D has been previously used as a measure of HRQoL in the context of depressive disorders in the national Finnish Health 2000 Survey (17) and as an outcome in a randomized clinical antidepressant pharmacotherapy trial (18).

Table 1 shows the characteristics of the 106 working-age primary care patients (77%) who completed the 15D at 5 years. In the SCID-I/P interviews at 5 years, 47% of the patients were clinically in full remission (mean 15D score 0.887) and 20% had MDE (mean 15D score 0.679).

**Table 1:** Socio-demographic and clinical characteristics of patients in Vantaa Primary Care Depression Study (*N* = 106).

Variable	At baseline		At 5 years	
	*N*	%	*N*	%
Male gender	21	19.8	21	19.8
Cohabiting	56	53.3	54	50.9
Employed	48	45.3	50	47.2
Anxiety disorder (any)	45	42.5	46	43.4
Generalized anxiety disorder	14	13.2	15	14,2
Panic disorder	7	6.6	17	16.0
Social phobia	18	17.0	14	13.2
Substance use disorder	13	12.3	19	17.9
Physical illness interferes with everyday life	46	43.4	58	54.7
	Mean	SD	Mean	SD
Hamilton Rating Scale for Depression (HAMD)	16.2	5.6	11.1	7.9
Age (years)	44.2	13.6		
Beck Depression Inventory (BDI)	19.0	10.4	14.1	11.1
Beck Anxiety Inventory (BAI)	17.2	12.6	13.1	12.2
Beck Hopelessness Scale (HS)	8.2	5.2	7.4	5.4
Perceived Social Support Scale—Revised (PSSS-R)	43.9	12.6	47.0	12.5
Social and Occupational Functioning Assessment Scale (SOFAS)	57.4	11.4	64.6	15.86
Health-related quality of life (HRQoL), 15D score	0.775	0.124	0.824	0.122

We defined remission from depression, in terms of the HRQoL, as when the 15D score reached the normal range of the general population. For this purpose, the 15D cut-off value was operationally defined as the population mean − 1 SD (approximately five sixths of the normal population scores above this threshold).

### 2.2. The Finnish health 2011 survey

As in our previous study (9), we used the 15D general population data from the National Health 2011 Survey. The invitation to take part in the Health 2011 Survey was sent to all surviving persons who had been included in the representative, two-stage stratified, random sample of the National Health 2000 Survey, aged 29+ in 2000. In addition, a new random sample of persons aged18–28 years was drawn (19). From this total Health 2011 sample those persons were selected, who were in the age range of patients at 5 years (*N* = 4,157), and this subsample was weighted to reflect the patients’ age distribution. The mean adjusted 15D score for the population was 0.931 (SD 0.073).

### 2.3. Statistical methods

Statistical analysis used receiver operating characteristic (ROC) analyses of the area under the curve (AUC). Optimal cut-off points for psychiatric scales in terms of HRQoL were defined by minimizing [(1-sensitivity) + (1-specificity)]. Patients below and above the median (52 years) were also separately examined to determine the influence of age. We also investigated Spearman’s rank correlation coefficients of individual items with the 15D total score.

## 3. Results

**Figure 2:**
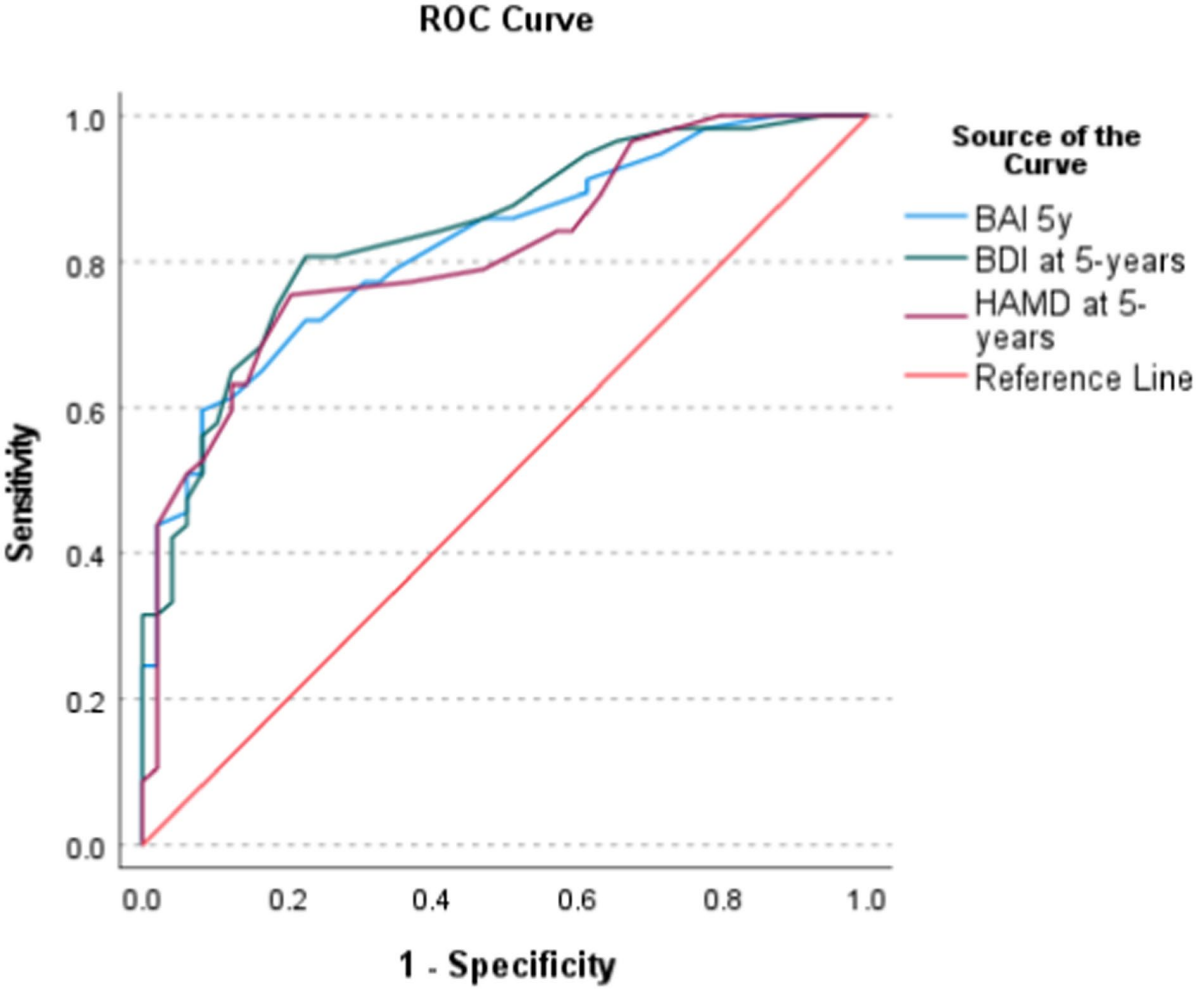
Receiver operating characteristic (ROC) curves for the three symptom scales and the 15D cut-off value—1 SD (0.858).

Table 2 presents the cut-off points of the HAMD, BDI and BAI compared to the HRQoL (15D score) of the age-standardized population, and Figure 2 the ROC curves. For the HAMD, BDI and BAI the AUC values were 0.813 (95% confidence interval. 0.732–0.894), 0.842 (0.768–0.916) and 0.821 (0.743–0.899), respectively (all *p* < 0.001).

**Table 2:** Depressive patients’ HAMD, BDI and BAI cut-off scores compared to HRQoL (15D) of general population (mean—1SD, and for comparison, mean), among all patients and age groups.

	15D score > mean 15D score—1 SD (0.858) of population 6	15D score > mean 15D score (0.931) of population	15D score > mean 15D score -1SD (0.838) of population aged >52 years	15D score > mean 15D score-1SD (0.877) of population aged <52 years
	*N* = 10	*N* = 106	*N* = 53	*N* = 53
HAMD	8.50	8.50	8.50	8.50
BDI	10.50	7.50	10.50	10.00
BAI	11.50	7.52	9.50	13.00

When the HAMD was ≤7, 91% of the patients were in full remission (DSM-IV) in the five-year interview. The proportion progressively increased when more stringent cut-offs were used (95% if HAMD ≤6; 97% if ≤5; 99% if ≤4, and 100% if ≤3). Similarly, when BDI was ≤6, 87% of the patients were in full remission, but the proportion increased when more stringent cut-offs were used (96% when BDI ≤5 or ≤ 4; 100% if ≤3).

All the BDI and most of the HAMD and BAI items significantly correlated with the 15D score. The strongest correlations (rs) in the HAMD were with the work and activities (rs = −0.625**) and depressed mood (rs = −0.547**) items. However, two items did not significantly correlate with the 15D – agitation (*p* = 0.412, rs = −0.080) and insight (*p* = 0.110, rs = −0.156). Their median was zero at both baseline and 5 years. The strongest correlations in the BDI were with the tiredness (rs = −0.659**) and indecisiveness (rs = −0.629**) items.

The strongest correlations in the BAI were those with the unsteadiness (rs = −0.578**) and feeling scared (rs = −0.457**) items. One BAI question (face flushing) did not significantly correlate with the 15D (*p* = 0.068, rs = −0.179). The median was zero at both baseline and 5 years.

## 4. Discussion

In this study, we evaluated the level of remission in depressive and anxiety symptoms required for HRQoL to reach the general population’s common range. We continued our earlier work (9) investigating the relationship between depression and HRQoL among primary care patients. We found that the optimal cut-off scores for remission were 8.5 in the HAMD, 10.5 in the BDI, and 11.5 in the BAI. Thus, they were quite close to the conventional cut-offs (HAMD <8; BDI and BAI < 10) of clinical remission, or caseness in screening.

This study had some major strengths. The cohort of depressive patients was based on screening, carefully diagnosed with semi-structured interviews including the evaluation of psychiatric comorbidity, and a longitudinal, five-year follow-up using life chart methodology (10, 11). Furthermore, the patients’ HRQoL was measured using the 15D and was comparable to that of a representative, age-standardized sample of the Finnish general population from the Health 2011 Study. However, some significant limitations require attention. The analyses were cross-sectional, and the number of patients participating in the five-year interviews modest. We also had to compare primary care patients in one city to a comparison group representing the whole of Finland. Therefore, the generalizability of our findings remains uncertain and needs to be replicated. However, our patients adequately represented the actual primary care patients in the City of Vantaa. Furthermore, different generic HRQoL instruments may produce different distributions of HRQoL scores for the same population (15, 16). Therefore, our findings are specific to the 15D and the other instruments should undergo the same analyses to establish the cut-off points based on their results. Finally, like all generic measures of HRQoL, the 15D items include feelings of depression and distress, causing some degree of circularity. However, there are phenomenological differences between variable daily feelings and depressive mood or anxiety symptoms as part of diagnostic criteria of mental disorder. They differ in terms of intensity, duration, controllability and associated distress or disability.

Remission from depression is usually defined as remission from depressive symptoms (2). In a systematic review, deZwart et al. (2) concluded that remission can best be defined as a less symptomatic state than previously assumed (HAMD ≤4 instead of ≤7) (2). However, this systematic review focused on clinical recovery, not HRQoL. We deliberately investigated remission from the of HRQoL perspective, which was measured independently from all psychiatric and somatic diagnoses and has been scarcely investigated. In a post-marketing paroxetine study, Sawamura et al. (4) re-evaluated the definition of remission on the HAMD, based on HRQoL measured by SF-36. They found that all HRQoL subscores negatively correlated with the HAMD scores, and a cut-off value for HAMD of ≤4 seemed the best indicator of remission (4). Our study was based on the premise that score above (mean 15D score—1 SD) of the general population is a valid cut-off, and the mean as an alternative cut-off (see Table 2) to be too stringent as a definition. Naturally, this methodological choice is debatable.

The symptom scales used are also likely to affect the results, as they differ in their content and psychometric properties. Depression scales differ considerably in both their content and responsiveness to change when depressive symptoms are mild. Fried (20) investigated the differences in the item content of seven common depression scales (including the HAMD and BDI), and found considerable differences in item content across instruments, substantial heterogeneity, and low overlap. Patients approaching clinical remission may also have heterogeneous and qualitatively different residual symptoms, so the numerical equivalence of the scores in the same scale may hide important qualitative differences (3). Furthermore, a study based on item response theory found the HAMD scale to have low precision and low responsiveness to change, particularly when depression was mild or moderate (21). More specifically, we found that two HAMD items (agitation and lack of insight) did not significantly correlate with the 15D score. Both these HAMD item scores were very low from baseline to the end of the follow-up. Our findings are consistent with the view that the way in which depression is measured may influence findings, and that the scales have significant differences.

A systematic review found physicians’ and patients’ perspectives of recovery from depression to differ significantly (8). Approximately half of the patients scoring ≤7 on the HAMD did not consider themselves to be in remission (3). However, we used both clinician- and self-rated symptom scales, with relatively concordant findings in terms of HRQoL. An important perspective to consider is that people in the general population have common illnesses and sources of distress, and that achieving a supernormal HRQoL may not be a realistic clinical aim.

Comorbid mental disorders are common among patients with depression in both the general population and primary care (10, 22), and their impact on the course of depression is mostly unfavorable (11). In our previous work (9), we found concurrent anxiety to significantly influence HRQoL. Here, we evaluated the role of remission from coexisting anxiety in HRQoL. We found that the correlations varied between the items in the BAI and the 15D, and that age had a significant effect.

In conclusion, remission greatly depends on the definitions and instruments chosen. However, from the HRQoL perspective, the conventional cut-offs of depression and anxiety measures appear to perform well.

## Data availability statement

Due to limitations posed by research permits and the Finnish legislation on data protection, datasets of this study are not publicly available. Requests to access the datasets should be directed to erkki.isometsa@hus.fi.

## Ethics statement

The studies involving human participants were reviewed and approved by Ethical Committee of The Helsinki and Uusimaa Hospital District. The patients/participants provided their written informed consent to participate in this study.

## Author contributions

KR has interviewed all the depressive cohort patients at the 5-year follow-up interviews, analyzed data, and drafted the manuscript. HS has undertaken analyses that compare the depressive cohort with the Finnish Health 2011 Survey, critically reviewed the manuscript, and is the developer of the 15D instrument. MV has screened and interviewed all the depressive cohort patients at baseline and interviewed them at six and 18 months, plus critically reviewed the manuscript. EI has designed the study, supervised writing, and critically reviewed the manuscript. All authors contributed to the article and approved the submitted version.

## Conflict of interest

The authors declare that the research was conducted in the absence of any commercial or financial relationships that could be construed as a potential conflict of interest.

## Publisher’s note

All claims expressed in this article are solely those of the authors and do not necessarily represent those of their affiliated organizations, or those of the publisher, the editors and the reviewers. Any product that may be evaluated in this article, or claim that may be made by its manufacturer, is not guaranteed or endorsed by the publisher.
